# Integration of metabolomics and other omics: from microbes to microbiome

**DOI:** 10.1007/s00253-024-13384-z

**Published:** 2024-12-19

**Authors:** Daewon Go, Gun-Hwi Yeon, Soo Jin Park, Yujin Lee, Hyun Gi Koh, Hyunjin Koo, Kyoung Heon Kim, Yong-Su Jin, Bong Hyun Sung, Jungyeon Kim

**Affiliations:** 1 Institute of Food Industrialization, Institutes of Green Bioscience and Technology, Seoul National University, Pyeongchang, Gangwon-Do 25354 Republic of Korea; 2 Synthetic Biology and Bioengineering Research Center, Korea Research Institute of Bioscience and Biotechnology (KRIBB), Daejeon, 34141 Republic of Korea; 3 Department of Biosystems and Bioengineering, KRIBB School of Biotechnology, Korea University of Science and Technology (UST), Daejeon, 34113 Republic of Korea; 4 Department of Inflammation and Immunity, Lerner Research Institute, Cleveland Clinic, Cleveland, OH 44195 USA; 5 Graduate School of International Agricultural Technology, Seoul National University, Pyeongchang-Gun, 25354 Gangwon-Do Republic of Korea; 6 Department of Biological and Chemical Engineering, Hongik University, Sejong, 30016 Republic of Korea; 7 Department of Biotechnology, Graduate School, Korea University, Seoul, 02841 Republic of Korea; 8 Carl R. Woese Institute for Genomic Biology, University of Illinois at Urbana-Champaign, Urbana, IL 61801 USA

**Keywords:** Metabolomics, Transcriptomics, Genomics, Microbiome, Multi-omics

## Abstract

**Abstract:**

Metabolomics is a cutting-edge omics technology that identifies metabolites in organisms and their environments and tracks their fluctuations. This field has been extensively utilized to elucidate previously unknown metabolic pathways and to identify the underlying causes of metabolic changes, given its direct association with phenotypic alterations. However, metabolomics inherently has limitations that can lead to false positives and false negatives. First, most metabolites function as intermediates in multiple biochemical reactions, making it challenging to pinpoint which specific reaction is responsible for the observed changes in metabolite levels. Consequently, metabolic processes that are anticipated to vary with metabolite concentrations may not exhibit significant changes, generating false positives. Second, the range of metabolites identified is contingent upon the analytical conditions employed. Until now, no analytical instrument or protocol has been developed that can capture all metabolites simultaneously. Therefore, some metabolites are changed but are not detected, generating false negatives. In this review, we offer a novel and systematic assessment of the limitations of omics technologies and propose-specific strategies to minimize false positives and false negatives through multi-omics approaches. Additionally, we provide examples of multi-omics applications in microbial metabolic engineering and host-microbiome interactions, helping other researchers gain a better understanding of these strategies.

**Key points:**

•* Metabolomics identifies metabolic shifts but has inherent false positive/negatives.*

•* Multi-omics approaches help overcome metabolomics’ inherent limitations.*

## Introduction

*Metabolomics* is the study of complex biological systems through large-scale quantification of metabolites to elucidate physiological changes (Li et al. 2020). With the development of analytical techniques such as nuclear magnetic resonance (NMR), chromatography, and mass spectrometry (MS), hundreds of metabolites can be analyzed simultaneously, leading to the development of metabolomics (Alseekh et al. 2021). Among the omics techniques, metabolomics is considered to be most directly related to phenotypes, as metabolites act as direct regulators of biological processes (Rinschen et al. 2019; Bauermeister et al. 2022). Conversely, other omics technologies such as genomics, transcriptomics, and proteomics provide information regarding abundance or mutations in DNA, RNA, and proteins that indirectly correlate with phenotypic changes (Rinschen et al. 2019). Therefore, metabolomics is widely used as one of the most powerful tools to discover unknown metabolic pathways and identify the causes of metabolic changes, such as metabolic bottlenecks in genetically modified microorganisms and host-microbe interactions (Jeon et al. 2023). For example, metabolic engineering of microorganisms often results in changes in cofactor metabolites, which can cause redox imbalances and lead to the production of byproduct metabolites (Kim et al. 2016, 2018). Metabolomics can provide insight into metabolic bottlenecks by tracking the levels of these cofactor or byproduct metabolites (Kim et al. 2022b). Also, microbiome and metabolomics studies have revealed a direct link between the occurrence of CDI (*Clostridium difficile* infection) and the conversion of primary bile acids to secondary bile acids by 7α-dehydroxylating gut bacteria (Kang et al. 2019). Many research groups are developing microbiome-based therapeutics to prevent CDI by targeting this mechanism (Mullish and Allegretti 2021).

**Fig. 1:**
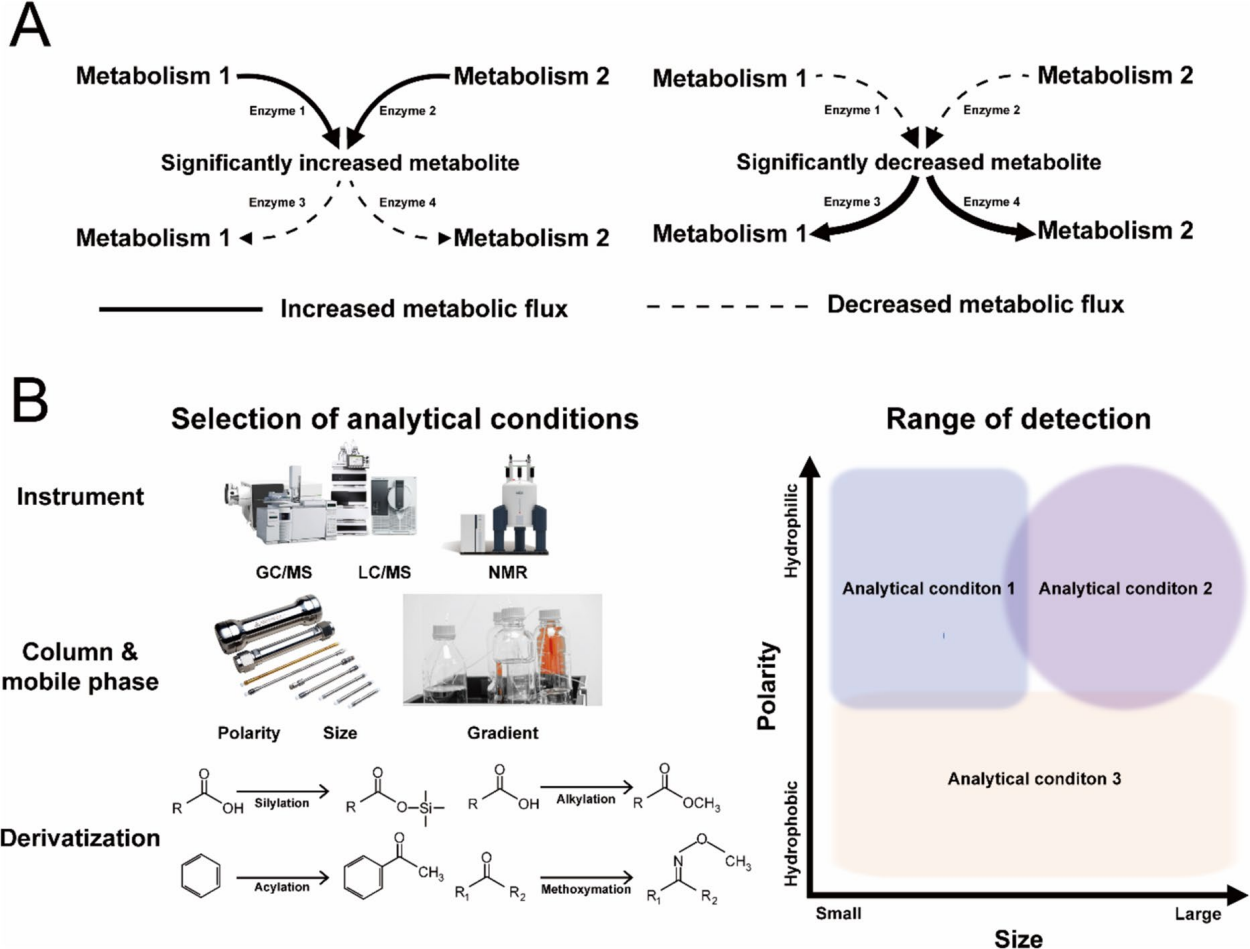
Inherent limitations of metabolomics. **A** Significant changes in metabolites due to the action of enzymes. **B** The detection range of metabolites according to the analytical conditions

Although metabolomics is a powerful tool that can be used for identifying the causes of phenotypic changes, it has inherent limitations. First, as each metabolite is a non-directional intermediate belonging to multiple biochemical reactions, it is difficult to infer which reaction causes metabolite changes (Johnson et al. 2016). As presented in Fig. 1A, significantly increased or significantly decreased metabolites belonged to metabolisms 1 and 2. This indicates that, even if a metabolite significantly increases or decreases compared to that in the control group, it remains unclear if the cause is metabolism 1 or metabolism 2. For example, glucose-6-phosphate (G6P) is not only an intermediate in glycolysis but also in gluconeogenesis and the pentose phosphate (PP) pathways. Therefore, even if G6P was significantly increased or decreased compared to that in the control group, it is difficult to infer which metabolic changes among the glycolysis, gluconeogenesis, and PP pathways induced changes in the abundance of G6P. Additionally, a significant increase in metabolites may result from increased activity of enzymes 1 and 2 or decreased activity of enzymes 3 and 4 (Fig. 1A). Similarly, a significant decrease in metabolites could be due to decreased activity of enzymes 1 and 2 or increased activity of enzymes 3 and 4 (Fig. 1A). Therefore, it is impossible to accurately track altered metabolism based solely on the concentrations of intermediate metabolites.

Second, there is no analytical methodology that can identify all metabolites simultaneously (Johnson et al. 2016; Alseekh et al. 2021). Metabolite analysis requires the selection of various analytical conditions, including instruments, columns, mobile phases, and derivatization methods (Alseekh et al. 2021). Additionally, the types and amounts of metabolites extracted vary depending on how they are extracted (Kim et al. 2020, 2022b). The detection range of metabolites depends on the combination of extraction methods and analysis conditions, and even with all methods combined, it is impossible to quantify all metabolites inside and outside of the cell using the current technology (Alseekh et al. 2021). As a result, the range of metabolites analyzed was much smaller than the actual range of metabolites present (Fig. 1B). For example, *Escherichia coli* or *Saccharomyces cerevisiae* are known to possess thousands of metabolites based on genome information (Feist et al. 2007; Kim et al. 2021; Oftadeh et al. 2021), but the numbers of identified metabolites are 700 and 500, respectively (Alseekh et al. 2021). As a result, a false-negative error occurs, in which certain metabolites are actually changed, but they are not detected and are regarded as unchanged.

To overcome these limitations, multi-omics approaches combined with metabolomics and other omics technologies have been undertaken. Genomics, transcriptomics, proteomics, and fluxomics provide information regarding enzymes (Picard et al. 2021; Wörheide et al. 2021; Pang et al. 2022), and combining them with metabolomics reveals the cause of altered metabolism and reduces false-positive errors. Additionally, as genomics, transcriptomics, and fluxomics provide information regarding all genes or transcripts or use models that are built based on them, all metabolic changes can be investigated (Wörheide et al. 2021; Rau et al. 2022). This can be used to identify metabolic changes associated with unidentified metabolites and to reduce false-negative errors.

In this review, we provide a novel and systematic analysis of the limitations of omics studies such as metabolomics, genomics, transcriptomics, and fluxomics, which have not been comprehensively addressed in previous research. In addition, we propose specific strategies to reduce false positives and false negatives through multi-omics approaches. Additionally, we present recent examples of multi-omics applications in microbial metabolic engineering and host-microbiome interactions to help other researchers better understand these approaches.

## Integration of metabolomics with genomics

Advances in high-throughput sequencing have made it possible to track mutations in every gene within an organism as well as to calculate the presence and relative abundance of specific microorganisms in a microbial population (Park and Kim 2016). Mutations in genes induce changes in the expression levels of transcripts and proteins or structural changes in proteins, and this greatly affects the amount of metabolites and changes the phenotype (Manzoni et al. 2018). Thus, whole-genome sequencing and metabolomics are frequently applied to a single organism to understand the metabolic changes caused by mutations in each gene and to identify the causes of physiological differences (Hou et al. 2020). For example, the genome of *Saccharomyces boulardii* is nearly identical to that of *Saccharomyces cerevisiae*, but *S. boulardii* is more tolerant to temperature and acid stress than *S. cerevisiae*. It also remains in the human intestine longer and exhibits various health benefits (Hossain et al. 2020; Kim et al. 2022a). In a previous study, Liu et al. reported that *S. boulardii* possesses a point mutation in *PGM2* that results in inefficient galactose metabolism and galactitol accumulation (Liu et al. 2018). Additionally, they demonstrated that replacing *PGM2* in *S. boulardii* with that of *S. cerevisiae* not only increased the efficiency of galactose metabolism but also decreased resistance to high temperatures, suggesting that the point mutations are related to temperature resistance (Liu et al. 2018). Similarly, the causes of phenotypic changes can be identified by investigating the correlation between genetic mutations and metabolomic changes in various organisms. Additionally, tools such as AlphaFold (Jumper et al. 2021) and RoseTTAFold (Baek and Baker 2022) can be used to predict structural changes in proteins synthesized by mutant genes and suggest the potential causes of metabolite accumulation and changes.

Advances in high-throughput sequencing technologies have made it possible to study microbiome (Sulit et al. 2023). Studies indicating that patients with diseases such as obesity (Sankararaman et al. 2023) and autism (Taniya et al. 2022) possess a specific microbiome structure have led to active research in this field to identify the relationship between microbiome structure and disease. However, in most cases, the mechanism by which a specific microbiome structure causes a specific disease has not yet been identified. By analyzing changes in the composition of the microbiome along with changes in the metabolome, mechanisms that induce physiological changes can be identified, and disease treatments can be devised (Hou et al. 2020; Kim et al. 2023a). For example, the mechanism of CDI has been revealed through investigations of the gut microbiome and metabolome (Buffie et al. 2015). In the normal intestine, bile acid 7α-dehydroxylating gut bacteria such as *Clostridium scindens* inhibit the growth of *C. difficile* by secreting tryptophan-derived antibiotics and converting the primary bile acids cholate and chenodeoxycholate into the secondary bile acids deoxycholate and lithocholate (Kang et al. 2019). However, in patients who received antibiotic treatment for various diseases, *C. difficile* proliferated due to the elimination of bile acid 7α-dehydroxylating gut bacteria, and they secreted toxins and caused CDI. Currently, the prevention and treatment of CDI are being developed based on these mechanisms (Guo et al. 2020), and studies are being actively conducted to identify the mechanisms of other diseases through microbiome analysis and metabolomics.

## Integration of metabolomics with transcriptomics

Transcriptomics is the study of the expression levels of a complete set of RNA transcripts in organisms (Dong and Chen 2013). Similar to genomics, it not only determines the direction of reactions but also relatively quantifies all messenger RNAs (Wang et al. 2009; Tian et al. 2022). However, transcript abundance is not often equal to protein function and is indirectly linked to the phenotype. Even highly altered transcripts may exhibit little correlation with phenotypic changes (Feder and Walser 2005). To overcome these shortcomings, transcriptomics is performed simultaneously with metabolomics. Metabolomics complements transcriptomics by offering more direct insights into phenotypic changes, while transcriptomics provides information about the metabolic pathways associated with unknown metabolites. For example, metabolomic and transcriptomic analyses were performed to investigate the basic metabolic activities of *Escherichia coli* Nissle 1917 (EcN) in the intestine (Kim et al. 2024). Interestingly, EcN metabolizes galactose, a mucin sugar, slowly under anaerobic conditions. Multi-omics analysis and gene modification revealed that the carbon flux of EcN that metabolizes galactose is introduced into the trehalose pathway to some extent, thus leading to intracellular trehalose accumulation and slow growth. In this study, 2768 transcripts exhibited significant changes between groups, and transcripts indirectly related to trehalose synthesis, such as stress resistance metabolism, were 50- to 100-fold higher in EcN-metabolizing galactose than in the other groups. In contrast, there were only four transcripts directly related to trehalose metabolism, and their expression in EcN that metabolizes galactose was 2- to threefold higher than that in the other groups. Nevertheless, based on metabolomics results, trehalose metabolism was suggested as the major cause of inefficient galactose metabolism. Similarly, transcriptomics and metabolomics can be applied to detect specific changes in metabolism by investigating the changes in metabolites associated with differentially expressed transcripts.

Integrated analysis of transcriptomics and metabolomics has been applied not only to single cells but also to the whole microbiome. Unlike 16 s rRNA sequencing that only examines taxonomic classification, meta-transcriptomics examines the expression of transcripts in a microbial community and can elucidate the expression of specific genes within a microbe, thus enabling functional annotation (Ojala et al. 2023). However, meta-transcriptomics still requires more information in the reference genome database, and it is necessary to reduce mis-annotation by improving the performance of software to assemble contigs and supercontigs correctly from short-read data (Aguiar-Pulido et al. 2016). Additionally, metatranscriptomics requires a method for identifying variables directly related to phenotypic changes among numerous microbiome transcripts. The integration of metatranscriptomics and metabolomics will play an important role in understanding how the microbiome causes or treats diseases by examining gene expression in specific microbes that change metabolites.

## Integration of metabolomics with metabolic flux balance analysis

Metabolic flux balance analysis is a computational and mathematical approach to analyzing the flux of metabolites through metabolic networks. In particular, genome-scale metabolic models (GEMs) can be used to predict the rates of growth or metabolite production in an organism by calculating the flow of metabolites. A GEM is a mass balance-based model consisting of metabolic reactions and metabolites within a target organism (Orth et al. 2010). By calculating the fluxes of the entire metabolism, including experimentally unidentified metabolites, GEMs can address the limitations of metabolomics, which focuses only on identified metabolites. Additionally, as GEM analysis is a computational simulation, it is very economical compared to other omics approaches that perform actual experiments. In previous studies, GEMs and metabolomics were used to elucidate the role of fucose in host-gut microbiome interactions. Fucose is a deoxyhexose that is continuously synthesized by humans in the form of fucosylated glycans and fucosyl-oligosaccharides and is delivered to the intestine from birth to death (Kim et al. 2023b). GEM and metabolomic results have demonstrated that gut microorganisms that metabolize fucose synthesize large amounts of short-chain fatty acids (SCFA) instead of biomass due to their low energy production efficiency (Kim et al. 2022a, 2023b; Cheong et al. 2022). As a result, human epithelial cells absorb most of the carbons used in fucose synthesis as SCFAs and use them as energy or signaling molecules, and thus, it was revealed that fucose plays an important role in host-microbiome interaction with its high energy conservation efficiency (Kim et al. 2023b). Similarly, Ottman et al. investigated the metabolism of mucin sugars, including fucose in *Akkermansia muciniphila*, which is an intestinal microorganism that suppresses obesity, through metabolite analysis and GEM (Ottman et al. 2017).

Recently, the Microbiome Modeling Toolbox, a part of the Cobra Tool Box, was developed to predict the balance of metabolic fluxes within the microbiome in addition to single cells (Heinken and Thiele 2022). The Microbiome Modeling toolbox enables the prediction of disease potential and microbial interactions such as mutualism, commensalism, and competition using 16 s rRNA and metagenomic sequencing data (Baldini et al. 2019; Heinken and Thiele 2022). To date, it has been used in more than 100 studies to investigate the relationship between the microbiome and various diseases such as inflammatory bowel disease (Heinken et al. 2021; Basile et al. 2023) and Parkinson’s disease (Baldini et al. 2020). The simultaneous application of genomics, metabolomics, and the Microbiome Modeling Toolbox will be more frequently exploited in the future. It will be of great help in identifying the causes of diseases by revealing changes in the microbiome and metabolome.

Although the GEM can accurately predict metabolic fluxes under specific conditions because its results are simulations, it is essential to tune and validate the model using real experiments. Kim et al. predicted that EcN metabolizes mucin sugars under anaerobic conditions and produces ethanol as its major extracellular product (Kim et al. 2021). However, previous study demonstrated that EcN metabolizes mucin sugars under anaerobic conditions and produces lactic acid but not ethanol (Kim et al. 2024). This is thought to be a prediction error, as both ethanol and lactic acid production are reactions that oxidize NADH to NAD^+^, and EcN contains genes involved in both metabolic pathways. Additionally, metabolic flux predictions by GEM are highly dependent upon external factors and thus possess limited use for calculating the metabolic activity of specific microorganisms in defined minimal media. As the metabolic flux of a microbiome composed of thousands of microbes possesses many variables, the difference between the calculated and actual values is even greater. Therefore, in the future, it will be necessary to improve in silico models so that metabolic flux can be accurately predicted by culturing individual strains or microbiomes in various media and examining the metabolites that are produced.

## Conclusions

Metabolomics is a powerful tool for uncovering the underlying causes of phenotypic changes. However, it has inherent limitations that lead to false positive and false negative errors. In this review, we examined the limitations of individual omics technologies and highlighted how integrating metabolomics with other omics approaches can overcome these challenges. We also discussed how multi-omics studies can be applied to identify metabolic bottlenecks for microbial metabolic engineering and to elucidate host-microbiome interactions. This review serves as a valuable resource for researchers, providing guidance on the effective use of multi-omics approaches. In the future, as omics technologies such as metametabolomics, metatranscriptomics, and the microbiome toolbox advance and are more widely applied in microbial community studies, systematic analyses and strategies for the multi-meta-omics studies will need to be suggested.
